# Hyperbaric Oxygen Improves Cerebral Ischemia/Reperfusion Injury in Rats Probably via Inhibition of Autophagy Triggered by the Downregulation of Hypoxia-Inducing Factor-1 Alpha

**DOI:** 10.1155/2021/6615685

**Published:** 2021-03-15

**Authors:** Cuiting Wang, Feng Niu, Ningna Ren, Xiaokun Wang, Hequan Zhong, Jie Zhu, Bing Li

**Affiliations:** ^1^Research Center for Clinical Medicine, Jinshan Hospital Affiliated to Fudan University, Shanghai 201508, China; ^2^The Institute of Neurology, The Academy of Integrative Medicine of Fudan University, Shanghai 200032, China; ^3^Department of Rehabilitation, Jinshan Hospital, Fudan University, Shanghai 201508, China; ^4^College of Acupuncture-Massage and Rehabilitation, Yunnan University of Traditional Chinese Medicine, 650500, China

## Abstract

Ischemic stroke, accompanied with high mortality and morbidity, may produce heavy economic burden to societies and families. Therefore, it is of great significance to explore effective therapies. Hyperbaric oxygen (HBO) is a noninvasive, nondrug treatment method that has been proved able to save ischemic penumbra by improving hypoxia, microcirculation, and metabolism and applied in various ischemic diseases. Herewith, we fully evaluated the effect of HBO on ischemic stroke and investigated its potential mechanism in the rat ischemia/reperfusion(I/R) model. Sixty Sprague-Dawley male rats were randomly divided into three groups—sham group, MCAO group, and MCAO+HBO group. In the latter two groups, the middle cerebral artery occlusion was performed (MCAO) for 2 hours, and then the occlusion was removed in order to establish the ischemic/reperfusion model. Subsequently, HBO was performed immediately after I/R (2 hours per day for 3 days). 72 hours after MCAO, the brain was dissected for our experiment. Finally, the data from three groups were analyzed by one-way analysis of variance (ANOVA) and followed by a Bonferroni test. In this article, we reported that HBO effectively reduced the infarction and edema and improved neurological functions to a certain extent. As shown by western blot analysis, HBO significantly reduced autophagy by regulating autophagy-related proteins (mTOR, p-mTOR, Atg13, LC3B II and LC3B II) in the hippocampus 72 hours after I/R, which was accompanied by inhibiting the expression of hypoxia inducible factor-1*α* (HIF-1*α*) in hippocampus. The results suggest that HBO may improve cerebral I/R injury, possibly via inhibiting HIF-1*α*, the upstream molecule of autophagy, and therefore, subsequently inhibiting autophagy in the rat model of ischemic stroke.

## 1. Introduction

According to the Global Burden of Disease (GBD 2010), cerebral stroke is the second most common cause of death and the third most common cause of disability-adjusted life years, which places heavy economic burdens on societies and families [1]. Among the various causes of stroke, the proportion of stroke caused by ischemia is as high as approximately 85% [2]. In ischemic stroke, a lack of blood flow can lead to excitotoxicity and necrotic cell death in the ischemic core of the brain within minutes [3]. An ischemic penumbra is found around the ischemic core, where the imbalance of energy supply and demand caused by the reduction of cerebral blood flow generates neuronal dysfunction and even damage; however, to some extent, this damage can be restored [4]. Moreover, it has also been observed that the restoration of blood flow may exacerbate cerebral injuries due to the activation of innate and adaptive immune responses and subsequent programmed cell death [5]. Consequently, the cells in the penumbra may progress to cell death, which in turn leads to the expansion of the ischemic core. In the case of further ischemic/reperfusion injuries, not only normal necrosis but also activation of autophagy in the cerebral ischemic penumbra has been observed [6]. Autophagy, a highly conserved metabolic mechanism of decomposition that maintains cellular homeostasis, is also termed type II programmed cell death to differentiate it from apoptosis [7]. The regulation of autophagy plays an important role in cell survival after ischemic cerebral stroke. The autophagy marker LC-3 II was detected in the penumbra during the acute phase of ischemic stroke [8]. With the overexpression of FKBP5 in oxygen-glucose deprivation and reoxygenation- (OGD/R-) injured neurons, a greater number of autophagic vacuoles were observed under an electron microscope, and increased LC-3B II was detected by western blotting [4]. In the middle cerebral artery occlusion (MCAO) mouse model, astrocyte-derived exosomes (AS-Exo) reduced infarct volume by inhibiting autophagy, and the protective effect of AS-Exo was counteracted by the use of rapamycin (an activator of autophagy) [9].

Regarding the treatment of ischemic cerebral stroke, it is of great significance to explore new intervention to achieve the combined or multiple therapies [10]. Hyperbaric oxygen (HBO), defined as breathing 100% O_2_ at pressures higher than the atmospheric pressure, is a noninvasive and nondrug treatment that has been widely used in neurological diseases, such as brain injury, cerebral palsy, and stroke [11]. The effectiveness of HBO is related to improving hypoxia, microcirculation, and metabolism and preventing further progression of deleterious secondary effects [12]. A large number of animal experiments and clinical trials have shown that HBO alleviates infarct size and oedema and reduces the permeability of the blood-brain barrier, which has a positive effect on ischemic stroke [13–16]. Spatial memory has been effectively improved in rats with ischemic hypoxic brain injury, and this improvement may be attributable to the preservation of damaged cortical and hippocampal cells [17]. Mechanistically, the effect of HBO may be related to the suppression of oxidative stress, inflammation, and cell death after ischemic stroke [18]. Therefore, HBO treatment may be a promising strategy in the treatment of ischemic cerebral stroke.

However, the exact mechanism of the therapeutic effect of HBO is still unclear. HBO therapy can effectively alleviate cerebral ischemia/reperfusion injury in rats, and this effect may be related to the inhibition of cerebral autophagy [13, 19, 20]. Hypoxia-inducing factor-1 alpha (HIF-1*α*) is the regulator of the response of mammalian cells to hypoxia and plays an important role in physiological hypoxia and pathological conditions [21]. HIF-1*α* is one of the HBO targets in ischemic diseases [22, 23]. Immunohistochemistry and immunoblotting revealed lowered HIF-1*α* protein levels in the ischemic hemisphere of HBO-treated mice [24]. Therefore, the purpose of this study was to verify the effect of HBO on MCAO rats and investigate the effect of HBO therapy on autophagy in ischemic stroke.

## 2. Materials and Methods

### 2.1. Animals and Groups

All animal experimental procedures were approved by the Animal Welfare & Ethics Committee of Shanghai Public Health Clinical Laboratory, Shanghai, China. Sixty male Sprague-Dawley rats (90 days old) purchased from Shanghai SLAC Laboratory Animal Co., Ltd. (China, SCXK (Shanghai) 2017-0005) were housed in a room with a controlled temperature of 24°C and 12-hour/12-hour light/dark cycles with free access to food and water. The weights of these rats were measured and ranged from 300 to 400 g. The rats were blocked by weight into three blocks of rats with similar weights, and then within each block, rats were randomly assigned to one of three experimental treatments in a completely randomized block design. That is, the rats (*N* = 60) were randomly divided into the sham control group, the MCAO group, and the MCAO+HBO group and three groups of 20.

### 2.2. Animal Model of Ischemic Stroke

Middle cerebral artery occlusion was performed according to Longa's method [25]. Briefly, the rats were anesthetized with isoflurane (5% induction and 2% maintenance). A heat-blunted silicone-coated 4-0 nylon suture was carefully inserted into the left common carotid artery incision, reached the carotid bifurcation, and advanced 18-20 mm along the internal carotid artery to the bidirectional middle cerebral artery (MCA). After 2 hours of occlusion, the suture was extracted. During the whole operation, the core temperature of the rats was maintained at 37 ± 0.5°C. Except for the MCA occlusion, the rats in the control group received similar treatment.

### 2.3. Hyperbaric Oxygen Therapy

Based on previous descriptions in other studies, we improved the hyperbaric oxygen therapy of MCAO rats [13, 15]. When middle cerebral artery occlusion was complete, the MCAO+HBO rats were immediately treated with 100% oxygen and 1.5 atmosphere absolute pressure (TAT) in hyperbaric oxygen for 2 hours, and the same treatment was performed once a day for three consecutive days. Simultaneously, the MCAO rats were exposed to normobaric medical air containing 22-78/O_2_-N_2_.

### 2.4. Histological Analysis

At 72 hours post-MCAO, the rats were sacrificed under deep anesthesia with 10% isoflurane. The dissected rat brains were frozen at -80°C and made into coronal sections that were stained with thionine. Finally, the volume of cerebral infarction was analyzed with Image Analysis Software [26]. In addition, the brain water content was detected by the discrepancy between the dry and wet weight when assessing cerebral oedema. Thus, the dissected brain was immediately weighed (i.e., wet weight), dried in an oven at 110°C, and weighed again (i.e., dry weight). The following expression can be used to describe this relationship: brain water content (%) = (wet weight − dry weight)/(wet weight) × 100% [27].

### 2.5. Neurological Deficit Scores

Researchers blinded to the experimental groups assessed the neurological severity scores of rats 3 days after MCAO as described previously [28]. The neurological grading scores ranged from 0 to 7 (0, normal, symmetrical movement without any abnormal signs; 1, right forelimb flexion during tail lift; 2, as described for 1, plus unsteady gait; 3, right anterior limb kept close to the breast during tail lift; 4, right turn when crawling; 5, right anterior claw pushed backward along with the signs described for 4; 6, repeating rotational motion with an immotile posterior right limb; 7, right recumbent position because of inability to support body).

### 2.6. Immunofluorescence

Immunofluorescence was carried out as previously described [13]. At 3 days post-MCAO, the rats were perfused with saline in the left ventricle, and then the brains were removed and fixed in 4% paraformaldehyde. Frozen sections of the hippocampus were permeated with 1% Triton X-100 to destroy the membranes and blocked with fetal bovine serum for 1 hour. Afterwards, the sections were first incubated with a primary antibody against HIF-1*α* (1 : 500, NB100-105, Novus) overnight at 4°C and were then incubated with a fluorescently labelled secondary antibody (Alexa Fluor 594-conjugated goat anti-mouse IgG) overnight at 4°C. Finally, the sections were stained with DAPI for observation under a fluorescence microscope.

### 2.7. Immunoblotting

The rats were sacrificed 72 hours after ischemia-reperfusion. The hippocampal tissues were first homogenized with ice-cold lysis buffer and then centrifuged at 12,000 rpm at 4°C for 20 min to obtain the supernatants. After measuring the protein concentration with the BCA detection kit, the protein samples were separated by SDS-polyacrylamide gels and then transferred to PVDF membranes. After being blocked with 5% bovine serum albumin for 2 hours, the membranes were incubated overnight at 4°C with primary antibodies against HIF-1*α* (1 : 500, NB100-105, Novus), mTOR, p-mTOR (1 : 1000, 2983S, 5536S, Cell Signaling Technology), Atg13, LC-3B, *β*-tubulin, and GAPDH (1 : 1000, 13273, 83506, 21465, 5174, Cell Signaling Technology). Horseradish peroxidase-conjugated antibody was used as the secondary antibody for a 2-hour incubation at room temperature. The enhanced chemiluminescence method was used to observe the immunoreactive bands, and the bands were analyzed using Image J.

### 2.8. Statistical Analysis

Data were expressed as the mean ± SEM. Differences among different groups were evaluated by one-way analysis of variance (ANOVA), followed by a LSD test. *p* < 0.05 was considered statistical significance. Before using ANOVA, we verified a normal distribution and homogeneity of variance of the data. The data were statistical analyses were performed using SPSS (25.0) and GraphPad Prism 8.0. Moreover, the person handling the data was blinded to the grouping situation.

## 3. Results

### 3.1. HBO Therapy Effectively Reduced Infarction and Edema

**Figure 1:**
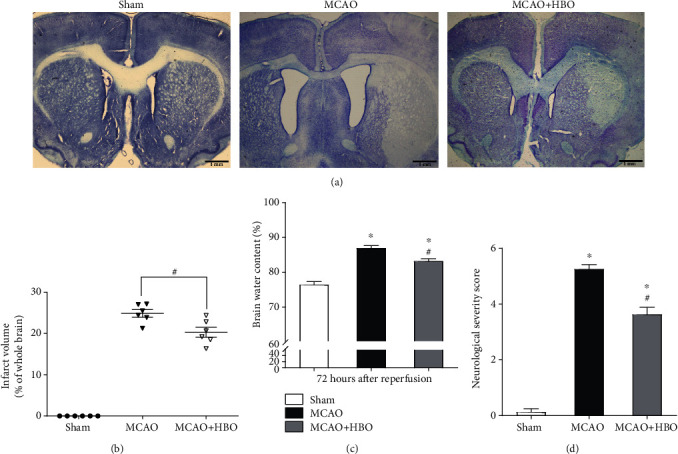
HBO effectively reduced infarct volume and brain edema and improved neurological functions. (a) The cerebral infarct volume in the sham, MCAO, and MCAO + HBO groups 72 hours after I/R injury indicated by thionine staining. The normal healthy region was stained blue. Scale bars, 1 mm. (b) Each point in the scatter plot represents the percentage of the infarct volume to the total brain volume (*n* = 6/group). (c) The percentage of the water content in the brains of the rats in the three different groups 72 hours after I/R injury (*n* = 6/group). (d) Neurological deficit assessment of different groups 72 hours after I/R injury. Data are expressed as the means ± SD from 8 individual rats in each group. ^∗^*p* < 0.05, compared with the sham group; #*p* < 0.05, compared with the MCAO group.

At 72 hours after ischemia/reperfusion, the infarct volume and brain water content of the three groups were compared by thionine staining and the wet/dry weight method. As shown in Figure 1(a), the normal brain tissues were stained blue by thionine, while the necrotic regions could not be stained and displayed a pale color.  Figure 1(b) shows that the infarct volume of the MCAO group (means ± SEM [24.875 ± 2.208], *p* < 0.001) and the MCAO+HBO group (means ± SEM [20.312 ± 2.940], *p* < 0.001) increased significantly compared with that of the sham group (means ± SEM [0.000 ± 0.000]). Moreover, the infarct volume of the MCAO+HBO group (*p* = 0.002) was significantly less than that of the MCAO group.  Figure 1(c) shows that the brain water content of the MCAO (means ± SEM [86.918 ± 1.780], *p* < 0.001) and MCAO+HBO groups (means ± SEM [83.117 ± 1.856], *p* < 0.001) was higher than that of the sham group (means ± SEM [76.380 ± 2.230]), while the brain water content of the MCAO+HBO group (*p* = 0.004) was lower than that of the MCAO group.

### 3.2. HBO Therapy Improved Neurological Function

In the neurological impairment score, a higher score represents more severe neurological impairment. As shown in Figure 1(d), neurological functions were significantly impaired in the MCAO (means ± SEM [5.250 ± 0.463], *p* < 0.001) and MCAO+HBO groups (means ± SEM [2.750 ± 0.707], *p* < 0.001) compared with the sham group (means ± SEM [0.000 ± 0.000]). Moreover, the MCAO+HBO group (*p* = 0.004) showed significant improvement in neurological function 72 hours after ischemia/reperfusion compared to that of the MCAO group.

### 3.3. HBO Therapy Inhibited Autophagy-Related Proteins in the Hippocampus

**Figure 2:**
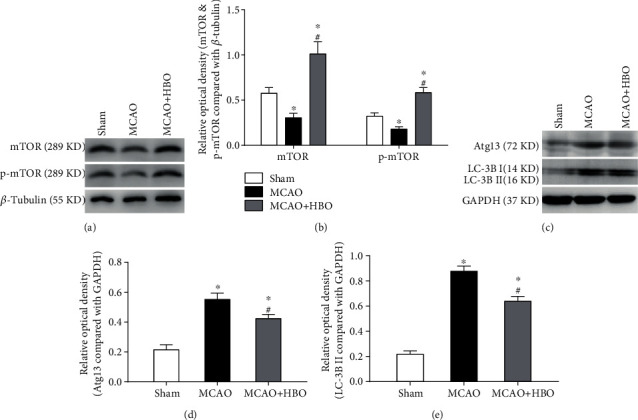
HBO inhibited the expression of autophagy-related molecules in the hippocampus following MCAO. (a, c) Western blots for mTOR, p-mTOR, Atg13, LC-3B I, and LC-3B II in the three groups. (b) Bar diagram showing the difference in the mTOR and p-mTOR expression in the ipsilateral cortex of the rats (*n* = 8/group). (d, e) Statistical analysis showing the expression of Atg13 and LC-3B II/LC3B-I in the ipsilateral cortex of the rats (*n* = 8/group). ^∗^*p* < 0.05, compared with the sham group; #*p* < 0.05, compared with the MCAO group.

The expression of autophagy-related proteins in the hippocampus was detected after continuous HBO treatment 72 hours after MCAO. As shown in Figures 2(a) and 2(b), compared with that in the sham operation group, the expression of mTOR and p-mTOR in the MCAO (*p* = 0.040, *p* = 0.016) and MCAO+HBO (*p* = 0.002, *p* < 0.001) groups decreased and increased, respectively, with an enhanced elevation in the MCAO+HBO group (*p* < 0.001, *p* < 0.001) compared with that of the MCAO group. In addition, ATG13 and LC-3B can also be detected as key molecules of autophagy. Western blot results suggested that the expression of ATG13 and LC-3B in the MCAO (*p* < 0.001, *p* < 0.001) and MCAO+HBO groups (*p* < 0.001, *p* < 0.001) was higher than that in the sham group, and the ATG13 and LC-3B expression was lower in the MCAO+HBO group (*p* = 0.013, *p* < 0.001) than in the MCAO group (Figures 2(c)–2(e)).

### 3.4. HBO Probably Decreased the Expression of HIF-1*α* in the Hippocampus after MCAO

**Figure 3:**
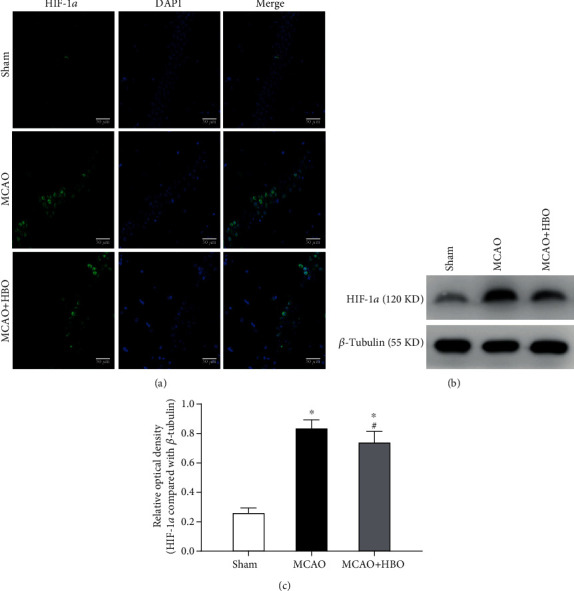
HBO suppressed the expression of HIF-1*α* in the hippocampus of MCAO-treated rats. (a) IF analysis showing the difference in the HIF-1*α* expression in the ipsilateral hippocampus of the rats. Scale bars, 50 *μ*m. (b) Western blots for the HIF-1*α* expression in each group. (c) Bar graph showing the quantitative analysis of HIF-1*α* (*n* = 8/group). ^∗^*p* < 0.05, compared with the sham group; #*p* < 0.05, compared with the MCAO group.

As shown in Figure 3(a), immunostaining of the brain sections demonstrated that HIF-1*α*-positive cells appeared in the hippocampus of the MCAO and MCAO+HBO rats but not in the sham control rats. In addition, the number of HIF-1*α*-positive cells in the hippocampus of the MCAO+HBO group was lower than that of the MCAO group. Furthermore, the quantity of HIF-1*α* in the hippocampus was verified by western blot. As shown in Figures 3(b) and 3(c), the expression of HIF-1*α* in the MCAO (*p* < 0.001) and MCAO+HBO (*p* < 0.001) groups was elevated compared with that in the sham group, whereas the HIF-1*α* expression was significantly lower in the MCAO+HBO (*p* = 0.004) group than in the MCAO group.

## 4. Discussion

Although reperfusion therapies such as intravenous thrombolysis and endovascular thrombectomy for ischemic stroke have made great progress, stroke is the second highest cause of death globally and a leading cause of disability [29, 30]. Therefore, the concept of combined or multiple therapies is meaningful for complicated stroke, and HBO therapy is one of the promising candidates [10]. HBO can restore/improve tissue oxygenation in ischemic organs as a physical therapeutic modality [31–33]. When it comes to the neuroprotective effect of HBO on ischemic stroke, it is under discussion. Although some papers have found that there is no therapeutic impact on patients after ischemic stroke [34], some other clinical data showed improved neurological outcomes in similar patients [14, 35, 36]. Therefore, it is of clinical significance to explore the therapeutic mechanism of HBO in stroke in consideration of the serious consequences of ischemic stroke in patients. Reduced blood flow and reperfusion can cause rapid necrosis of the ischemic core and delayed cell death in the ischemic penumbra, and it is meaningful to intervene in the ischemia/reperfusion process in the penumbra area through effective treatment, such as early thrombolysis, to rescue the ischemic penumbra [37]. In addition, the basic principle of HBO intervention is to increase the oxygenation of the ischemic penumbra region [38]. Our research demonstrates the significant protective effects of HBO treatment at an early stage in rats with temporary middle cerebral artery occlusion, including improvements in both neurological structures and functions. In line with previous reports [14, 18, 39], our research also demonstrated that the infarct volumes, brain edema, and neurological function scores of MCAO rats were significantly decreased when HBO was applied for 2 hours/day and lasted for 3 consecutive days after MCAO, which highlighted the necessity of early interference with HBO in ischemic stroke.

Although HBO improves the prognosis of stroke, the mechanism is still unclear. It has been reported that apoptosis and autophagy are the main types of cell death in the potentially salvageable ischemic penumbra [40]. Autophagy is a cellular process by which lysosomes degrade damaged organelles, certain pathogens, and cytoplasmic proteins to maintain intercellular homeostasis. Within 48 h after ischemic stroke, the expression of LC-3 II in the penumbra of MCAO rats was significantly higher than that in the brain tissue of rats without MCAO treatment [8]. Some reports have shown that the application of autophagy inhibitors significantly decreases brain damage by ischemia/reperfusion, whereas the activation of autophagy exacerbates injury [41, 42]. In addition, the ratio of the autophagy proteins LC-3B II/LC-3B I decreased when HBO treatment was performed in the MCAO rat model [13]. Based on these findings, we proposed that hyperbaric oxygen may reduce cerebral infarction by reducing autophagy in ischemic areas. The experimental results from our study demonstrated that HBO may improve brain injury insulted I/R by inhibiting autophagy. The alterations in the levels of key protein molecules in autophagy (mTOR, p-mTOR, Atg13, LC-3B II) brought about by hyperbaric oxygen confirmed the effects of HBO on autophagy, which revealed the role of autophagy in cerebral ischemic stroke and showed that the therapeutic effects of HBO may act via suppression of autophagy.

Although it remains unclear how HBO induces antiautophagy effects, some hypotheses have been proposed. HIF-1*α* is considered to be the key player in cellular adaptation and survival under hypoxic conditions, and it is used as a way of increasing oxygen pressure in the environment to relieve several symptoms associated with hypoxia [43]. One study has confirmed that the HIF-1 pathway was involved in hypoxia-induced autophagy in cardiomyocytes [44]. Considering these characteristics, it is easy to hypothesize that there is a correlation between the therapeutic effects of HBO on ischemic stroke and HIF-1*α*. Sun et al. confirmed that HBO decreased HIF-1*α* in the ischemic hemisphere [24]. In their study, the overexpression of HIF-1*α* enhanced the cleavage and recruitment of autophagosomes by LC-3 and the expression of autophagy-related molecules, while the knockdown of HIF-1*α* weakened the expression of LC-3. Similarly, Yang et. al argued that autophagic cell death caused by hypoxia in microglia was mediated via upregulation of hypoxia-inducible factor-1*α* [45]. Additionally, other study reported that dexmedetomidine inhibited autophagy by upregulating the expression of HIF-1*α* [46]. Previous studies have shown that HIF-1*α* is involved with the regulation of autophagy. Our results study demonstrated that the HIF-1*α* expression in the hippocampus of MCAO rats was reduced by HBO treatment. Meanwhile, the autophagy of brain after I/R was inhibited and further improved the cerebral damage. However, how HBO affect the cerebral expression of HIF-1*α* on cerebral I/R injury is our focus in the following study, which is significant to explore the molecular mechanism of HBO on improving I/R injury.

Although HBO has some applications in the clinical treatment of stroke, the mechanism of HBO on stroke is not clear. Besides, the time window, dosage, and side effects of HBO after stroke need to be resolved [47]. Therefore, its clinical application and promotion are limited. However, relevant experimental studies have shown that HBO is an attractive and promising candidate for the treatment of stroke. Meanwhile, the US Food and Drug Administration declared artery occlusion as one of the 13 specific indications for HBO therapy in August 2013 [47]. Our research has suggested that the protective effect of HBO on I/R rats is correlated to inhibition autophagy due to downregulation of HIF-1*α*. Some studies have discovered that HBO may affect the level of oxygen free radicals and further affect the expression of HIF-1*α* [22, 48]. Our next research focuses on whether HBO affects the oxidative response in the brain, which has a further impact on HIF-1*α* and autophagy. Therefore, it is significant to explore the exact underlying mechanisms of HBO therapy and further promote the clinical application of HBO for stoke.

## 5. Conclusions

In summary, early HBO treatment can reduce brain damage caused by ischemia/reperfusion, potentially by inhibiting autophagy and its upstream molecule HIF-1*α* to prevent neuronal death in the ischemic penumbra. This mechanism may be one of the important pathways by which HBO induces neurological protection and improves neurological function.

## Data Availability

The data used to support the findings of this study are available from the corresponding author upon request.

## Supplementary Materials

Supplementary Materials See Table S1-S2 in the Supporting Material for the mean, SEM, and *p* value of all data in the study.
