# Supplementary material for: Hyperbaric Oxygen Improves Cerebral Ischemia/Reperfusion Injury in Rats Probably via Inhibition of Autophagy Triggered by the Downregulation of Hypoxia-Inducing Factor-1 Alpha
Source: Biomed Res Int. 2021 Mar 15;2021:6615685. doi: 10.1155/2021/6615685 (PMC7987430; doi:10.1155/2021/6615685)
Supplement: Supplementary Materials — See Table S1-S2 in the Supporting Material for the mean, SEM, and p value of all data in the study. [file 6615685.f1.docx]

|  | N | **Means ±SEM** | | | **F Value** | **p Value** |
| --- | --- | --- | --- | --- | --- | --- |
|  |  | Sham | MCAO | MCAO+HBO |  |  |
| infarct volume | 6 | 0.000±0.000 | 24.875±2.208 | 20.312±2.940 | 233.549 | <0.001 |
| brain water content | 6 | 76.380±2.230 | 86.918±1.780 | 83.117±1.856 | 274.734 | <0.001 |
| neurological severity score | 8 | 0.000±0.000 | 5.250±0.463 | 2.750±0.707 | 231.700 | <0.001 |
| mTOR | 8 | 0.580±0.167 | 0.308±0.138 | 1.015±0.372 | 16.493 | <0.001 |
| pmTOR | 8 | 0.325±0.096 | 0.181±0.066 | 0.586±0.149 | 28.215 | <0.001 |
| Atg13 | 8 | 0.216±0.09 | 0.553±0.115 | 0.425±0.07 | 25.777 | <0.001 |
| LC-3B II | 8 | 0.219±0.070 | 0.878±0.114 | 0.641±0.101 | 95.353 | <0.001 |
| HIF-1α | 8 | 0.258±0.099 | 0.909±0.139 | 0.738±0.077 | 80.621 | <0.001 |

Table S1: one-way analysis of variance

Note: Sixty Sprague-Dawley male rats were randomly divided into three groups of 21, including Sham group, MCAO group and MCAO+HBO group. In the infarct volume experiment and the immunostaining of HIF-1α in the brain sections, eighteen rats(n=6) were utilized. The brain water content experiment is the same as the infarct volume experiments in the number and grouping of rats. The neurological severity score and the level of these proteins(mTOR, pmTOR, Atg13, LC-3B II and HIF-1α) expression experiments were conducted in same animals that were three groups of twenty-four.

Table S2: p-values for post-hoc pairwise comparisons

|  | **p-values for post-hoc pairwise comparisons** | | |
| --- | --- | --- | --- |
|  | Sham vs MCAO | Sham vs MCAO+HBO | MCAO vs MCAO+HBO |
| infarct volume | 0.000 | 0.000 | 0.002 |
| brain water content | 0.000 | 0.000 | 0.004 |
| neurological severity score | 0.000 | 0.000 | 0.004 |
| mTOR | 0.040 | 0.002 | <0.001 |
| pmTOR | 0.016 | 0.000 | <0.001 |
| Atg13 | 0.000 | 0.000 | 0.013 |
| LC3B II | 0.000 | 0.000 | <0.001 |
| HIF-1α | 0.000 | 0.000 | 0.004 |
